# Supplementary material for: Mathematical modeling indicates that regulatory inhibition of CD8+ T cell cytotoxicity can limit efficacy of IL-15 immunotherapy in cases of high pre-treatment SIV viral load
Source: PLoS Comput Biol. 2023 Aug 24;19(8):e1011425. doi: 10.1371/journal.pcbi.1011425 (PMC10482305; doi:10.1371/journal.pcbi.1011425)
Supplement: S1 Text — (PDF) [file pcbi.1011425.s001.pdf]

S1 Text – Supplement for:

Mathematical modeling indicates that regulatory inhibition of CD8⁺ T cell cytotoxicity can limit efficacy of IL-15 immunotherapy in cases of high pre-treatment SIV viral load

Contents

Contents ... 1
Model Comparison ... 2
Alternative Model $\boldsymbol{\alpha}$: Shorter SIV-specific $CD8^+$ T cell expansion ... 4
Alternative Model $\boldsymbol{\beta}$: Convolved SIV-specific and non-SIV-specific $CD8^+$ T cells ... 6
Alternative Model $\boldsymbol{\gamma}$: Model $\boldsymbol{\beta}$ without programmed expansion ... 8
Alternative Model $\boldsymbol{\delta}$: Non-SIV-specific cytotoxicity ... 10
Supplemental Methods ... 12
Parameter Space Discussion and Bayesian Distributions ... 12
Parameter Calculation ... 13
Fitting to single cohorts (for S3 Fig) ... 16
Comparison to other non-human primate cohorts (for S4 Fig) ... 16
Reference ... 17

All data and code required to replicate this work (in MATLAB R2018b or later) can be download at the following link:

https://github.itap.purdue.edu/ElsjePienaarGroup/N803_SIV_Model/releases/tag/v2.0

Model Comparison

In order to limit model complexity while satisfactorily replicating the NHP data, many model variants were calibrated to the data before the final model chosen (Eq. 1-11 in the main text). Four of these alternative models are discussed here. Description of the changes for each model α, β, γ, and δ are summarized in the sections below. Fig A(a) compares the negative log-likelihood (NLL, Eq. 12), which is a measure of model error with respect data. Fig A(b) compares the Akaike Information Criterion (Eq. S1), which incorporates a penalty term for number of model parameters. The model defined by Eq. 1-11 has the lowest values for both metrics, which motivates its selection over alternatives.

Eq. S1 $$\mathrm{AIC} = 2\mathrm{NLL} + 2\frac{(n_\theta + n_y)n_t}{n_t - n_\theta - n_y - 1}$$

Equation S1 is adapted from the AIC for multivariate regression with small data sets [1]. Here, we consider the number of parameters (n_θ) to be number of values required for the parameter calculation functions (see Fig 2 in the main text). In other words, we consider the assumption of starting the system of ordinary differential equations from two different steady-states with one set of constants to be an integral part of the model. For Eq. 1-11 and for models $\alpha, \beta, \gamma, \delta$ the number of parameters is n_θ=27,27,23,25,28, respectively. The formula also considers the number of response variables (n_y=4) and the number of data points per response variable (n_t=553/4).

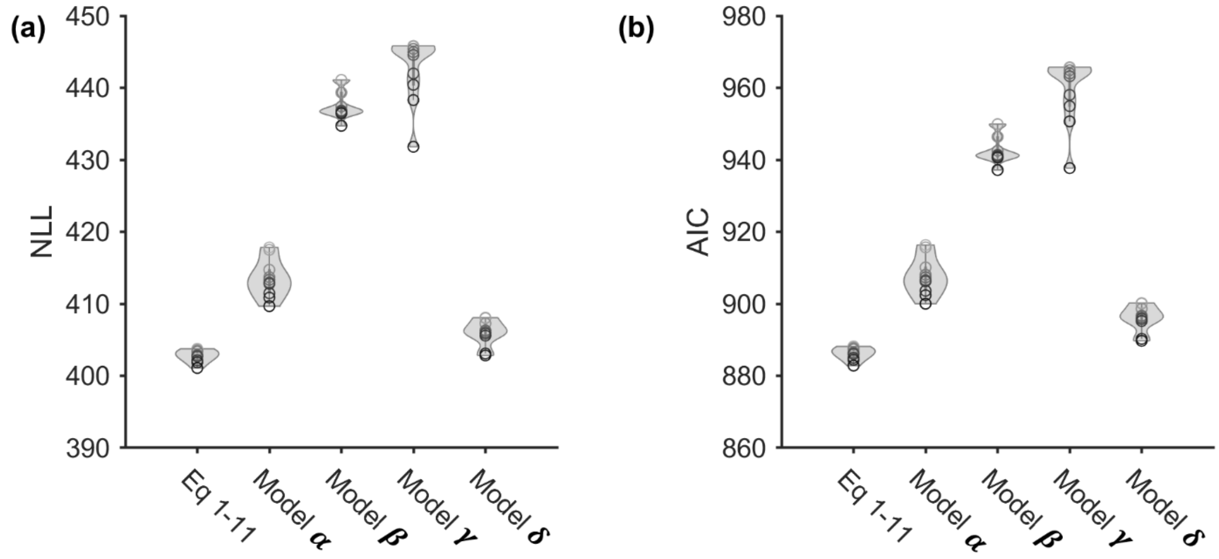

Fig A. Comparison of chosen model to alternative models using NLL and AIC. Compared are the best 10 results from the multi-start local-search for the final model (Eq. 1-11) and for alternative models $\alpha, \beta, \gamma, \delta$. The Negative Log Likelihood (NLL, Eq. 12) is a measure of model goodness-of-fit, while the Akaike Information Criterion (AIC, Eq. S1) is a model comparison metric that included a penalty for the number of model parameters. This parameter count is 27 for the final model, based on the number of values required to define all model constants and initial conditions for both cohorts based on the assumption of pre-treatment steady states for both cohorts. For models $\alpha, \beta, \gamma, \delta$ the number of parameters is 27,23,25,28, respectively.

Alternative Model α: Shorter SIV-specific CD8$^+$ T cell expansion

This model tests whether or not the number of divisions after SIV-specific CD8$^+$ T cell expansion can be reduced. In the final model, these cells divide 8 times after activation, in accordance with other models of T cell expansion [2, 3]. Here, in model α, these cells only divide 6 times after activation. Thus, Eq. 2-5 in the final model are replaced by Eq. S2-S5.

Eq. S2 $$S_0' = \frac{pS_0}{1+\varphi R_2}\left(\frac{S_{50}}{S_{50}+\sum_{i=0}^{6} S_i}\right)\left(1+\frac{\rho C}{C_{50}+C}\right) - dS_0 - \frac{\alpha_S S_0}{1+\zeta_S R_2}\left(\frac{V}{V+V_{50,S}}\right)\left(1+\frac{\alpha_S C}{C_{50}+C}\right) + m_S S_6$$

Eq. S3 $$S_1' = 2\frac{\alpha_S S_0}{1+\zeta_S R_2}\left(\frac{V}{V+V_{50,S}}\right)\left(1+\frac{\alpha_S C}{C_{50}+C}\right) - d_A S_1 - p_A S_1$$

Eq. S4 $$S_i' = 2p_A S_{i-1} - d_A S_i - p_A S_i \quad \text{where } i \in 2,3,4,5$$

Eq. S5 $$S_6' = 2p_A S_5 - d_A S_6 - m_S S_6$$

While model α replicates generally similar viral load behavior (Fig B(a,d)), the expansion for CD8$^+$ T cells in cohort 1 is not as strong (Fig B(b,e)). The additional two divisions in the final model allow it to match the data more closely, as shown by the NLL (Fig A(a)). These models otherwise use the same number of parameters.

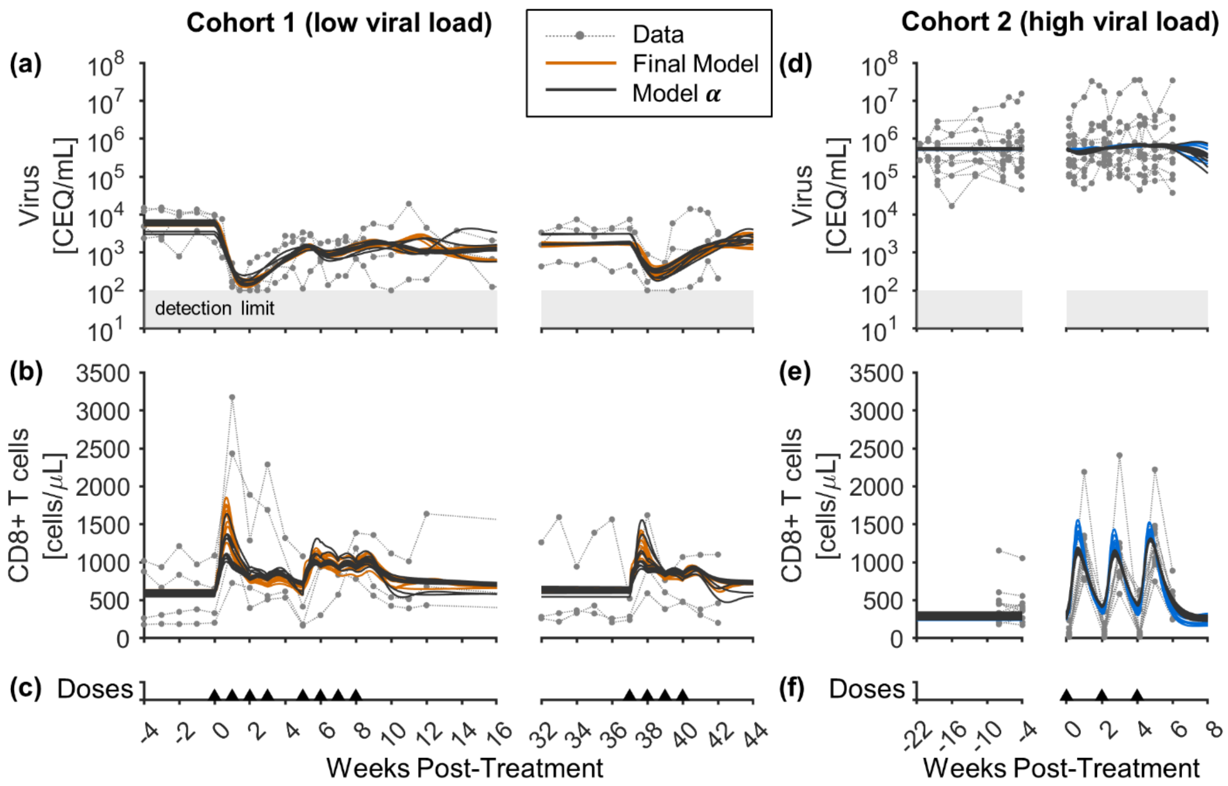

Fig B. Comparison of Model α to Final Model. Model α was calibrated to (a,d) virus in the plasma and (b,e) $CD8^+$ T cells in the peripheral blood from two different Simian Immunodeficiency Virus (SIV) cohorts. The top 10 results (lowest NLL, Eq. 12) from the multi-start local-search algorithm are compared for the final model (Eq. 1-11, orange/blue) and for the model α (black). The gray shaded region indicates the lower limit of detection for the viral assay (100 CEQ/mL). Panels (c,f) show timing of 0.1 mg/kg subcutaneous doses of N-803.

Alternative Model β: Convolved SIV-specific and non-SIV-specific CD8⁺ T cells

This model tests whether SIV-specific CD8⁺ T cells and non-SIV-specific cells can be combined. In the final model, SIV-specific CD8⁺ T cell undergo programmed expansion after activation, while non-SIV-specific CD8⁺ T cells divide only once. Here, in model β, we combine these two groups into one CD8⁺ T cell group, denoted by E, that divides 4 times after activation. Thus, Eq. 2-7 in the final model are replaced by Eq. S6-S9.

Eq. S6 $$E_0' = \frac{pE_0}{1+\varphi R_2}\left(\frac{E_{50}}{E_{50}+\sum_{i=0}^{4} E_i}\right)\left(1+\frac{\rho C}{C_{50}+C}\right) - dE_0 - \frac{\alpha E_0}{1+\zeta R_2}\left(\frac{V}{V+V_{50,E}}\right)\left(1+\frac{\alpha C}{C_{50}+C}\right) + mE_4$$

Eq. S7 $$E_1' = 2\frac{\alpha E_0}{1+\zeta R_2}\left(\frac{V}{V+V_{50,E}}\right)\left(1+\frac{\alpha C}{C_{50}+C}\right) - d_A E_1 - p_A E_1$$

Eq. S8 $$E_i' = 2p_A E_{i-1} - d_A E_i - p_A E_i \quad \text{where } i \in 2,3$$

Eq. S9 $$E_4' = 2p_A E_3 - d_A E_4 - mE_4$$

Furthermore, in this model, variables governing regulation generations are separated from those governing CD8⁺ T cell activation, so equation S10 replaces Eq. 10. This introduces a new parameter, ς, which was fitted within the boundaries given in Table A. This table also shows parameter boundaries that were widened from those given in Table 2.

Eq. S10 $$R_1' = s\left(\frac{V}{V+V_{50,R}} + \frac{\varsigma C}{C_{50}+C}\right) - d_R R_1$$

Similarly to model α, model β does not replicate the CD8⁺ T cell responses of both cohorts as well as the final model. In this case, it is the cohort 2 response where the models differ (Fig C(e)), where model β is not replicating the degree of cell expansion for this cohort. Many variations of models where SIV-specific CD8⁺ T cells and non-SIV-specific cells are combined were evaluated, and these performed as well or worse than model β.

Table A. Changes to parameter space for Model β

Initial Condition or Constant	Symbol	Value	Units
Reversion rate constant	m	(0.004, 0.4)	/day
Proliferation rate constant (active CD8⁺ T cells)	p_A	(0.5, 4)	/day
N-803 stimulation factor for regulation generation	ς	(0.01, 100)	

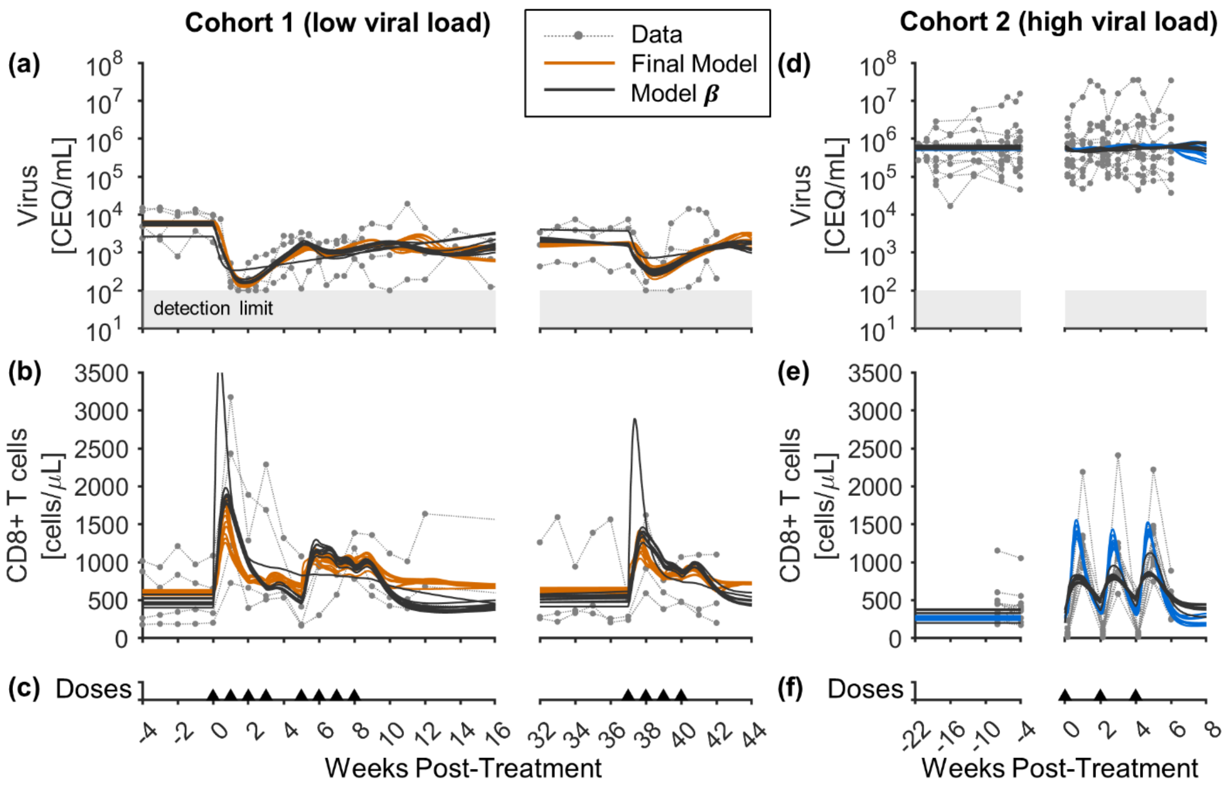

Fig C. Comparison of Model β to Final Model. Model β was calibrated to (a,d) virus in the plasma and (b,e) CD8$^+$ T cells in the peripheral blood from two different Simian Immunodeficiency Virus (SIV) cohorts. The top 10 results (lowest NLL, Eq. 12) from the multi-start local-search algorithm are compared for the final model (Eq. 1-11, orange/blue) and for the model β (black). The gray shaded region indicates the lower limit of detection for the viral assay (100 CEQ/mL). Panels (c,f) show timing of 0.1 mg/kg subcutaneous doses of N-803.

Alternative Model γ: Model $\boldsymbol{\beta}$ without programmed expansion

This model tests an alternative to model β where programmed expansion is replaced with a proliferation term of similar form to the term describing resting CD8[+] T cell proliferation. These terms are also reformed to use a base proliferation rate (p,p_A) and maximum proliferation rate (p_{max},$p_{A,max}$), rather than a base rate and stimulation factor (p,ρ in Eq. 2,6). This is to ensure that active cells do not proliferate too much faster than the experimentally measured division time of 6 hours for activated CD8[+] T cells [4]. In model γ, Eq. 2-7 in the final model are replaced by Eq. S11-S12.

Eq. S11 $$E_0' = \frac{E_0}{1+\varphi R_2}\left(\frac{E_{50}}{E_{50}+E_0+E_1}\right)\left(\frac{pC_{50}+p_{max}C}{C_{50}+C}\right) - dE_0 - \frac{aE_0}{1+\zeta R_2}\left(\frac{V}{V+V_{50,E}}\right)\left(1+\frac{\alpha C}{C_{50}+C}\right) + mE_1$$

Eq. S12 $$E_1' = \frac{E_1}{1+\varphi_A R_2}\left(\frac{E_{50}}{E_{50}+E_0+E_1}\right)\left(\frac{p_A C_{50}+p_{A,max}C}{C_{50}+C}\right) - d_A E_1 + 2\frac{aE_0}{1+\zeta R_2}\left(\frac{V}{V+V_{50,E}}\right)\left(1+\frac{\alpha C}{C_{50}+C}\right) - mE_1$$

As with model β, variables governing regulation generations are separated from those governing CD8[+] T cell activation, so Eq. S10 (previous section) replaces Eq. 10. Changes to the parameter space for model γ are summarized in Table B. Base proliferation rate for active CD8[+] T cells (p_A) is interpolated between the resting proliferation (p) and maximum active proliferation rate ($p_{A,max}$), Eq. S13.

Eq. S13 $$p_A = p + p_n\left(p_{A,max} - p\right) \qquad \text{where } p_n \in [0.1,100] \text{ (Table B)}$$

While this model was capable of qualitative behavior similar to the final model and to the data, the quantitative fit was lacking. There was a smaller viral load drop in cohort 1 (Fig D(a)) and a smaller CD8+ T cell expansion in cohort 2 (Fig D(e)).

Table B. Changes to parameter space for Model $\boldsymbol{\gamma}$

Initial Condition or Constant	Symbol	Value	Units
Reversion rate constant	m	(0.05, 0.5)	/day
Normalized active proliferation rate constant	p_n	(0.01, 1)	/day
Maximum resting proliferation rate constant	p_{max}	(0.02, 2)	/day
Maximum active proliferation rate constant	$p_{A,max}$	(0.4, 4)	/day
N-803 stimulation factor for regulation generation	ς	(0.1, 100)	

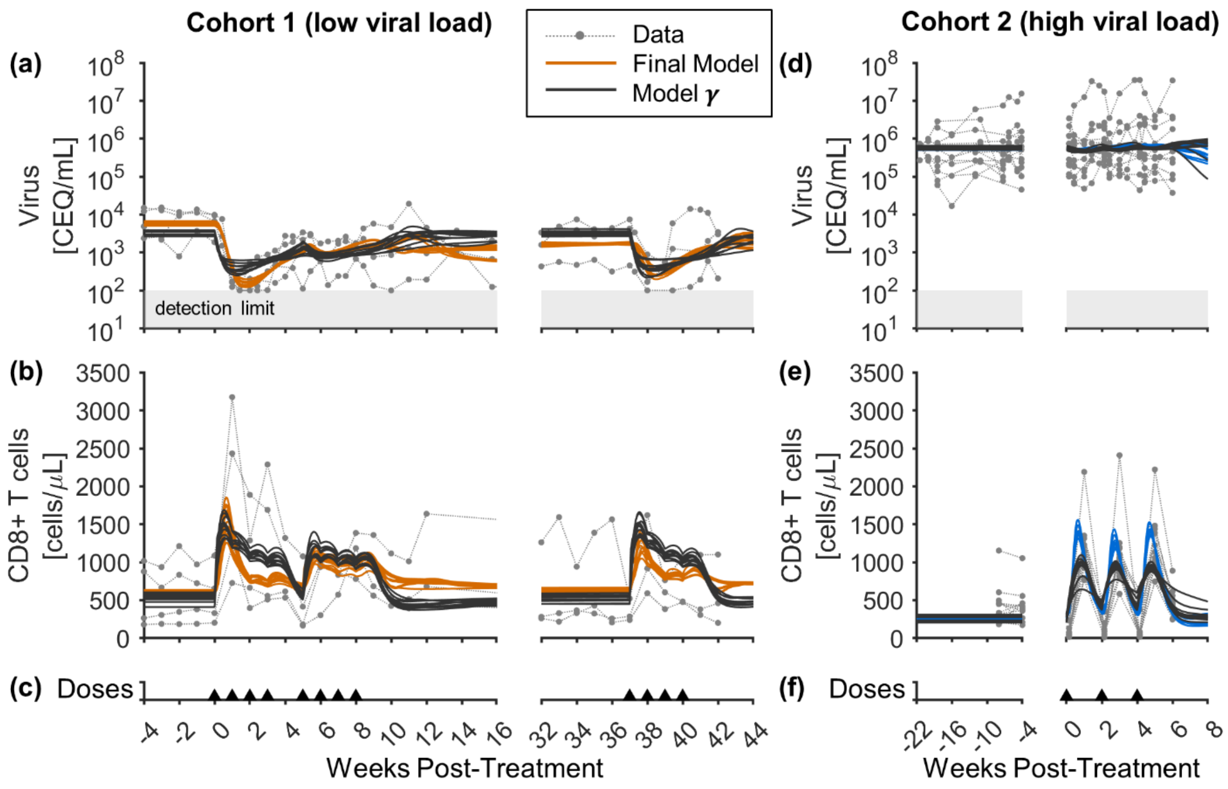

Fig D. Comparison of Model γ to Final Model. Model γ was calibrated to (a,d) virus in the plasma and (b,e) CD8$^+$ T cells in the peripheral blood from two different Simian Immunodeficiency Virus (SIV) cohorts. The top 10 results (lowest NLL, Eq. 12) from the multi-start local-search algorithm are compared for the final model (Eq. 1-11, orange/blue) and for the model γ (black). The gray shaded region indicates the lower limit of detection for the viral assay (100 CEQ/mL). Panels (c,f) show timing of 0.1 mg/kg subcutaneous doses of N-803.

Alternative Model δ: Non-SIV-specific cytotoxicity

This model tests whether it is necessary to assume some amount of viral suppression via killing of infected cells by non-SIV-specific CD8$^+$ T cells. While these cells are capable of non-specific cytotoxicity [5], it may not be necessary to include that in the model. Model δ evaluates this necessity by adding a non-specific killing term to Eq. 1. We assume that this non-specific killing rate is lower than the specific killing rate (Table C).

Eq. S14 $$V' = qV - \frac{g_S \sum_{i=1}^{8} S_i}{1+\lambda R_2} V - \frac{g_N N_1}{1+\lambda R_2} V$$

This model yielded nearly identical results to the final model (Fig E), demonstrating that including of non-specific cytotoxic action is not necessary for this model and data set. Thus, we chose to neglect this additional complexity to improve identifiability of the model.

Table C. Changes to parameter space for Model δ

Initial Condition or Constant	Symbol	Value
Killing rate ratio	g_N/g_S	(0.01, 1)

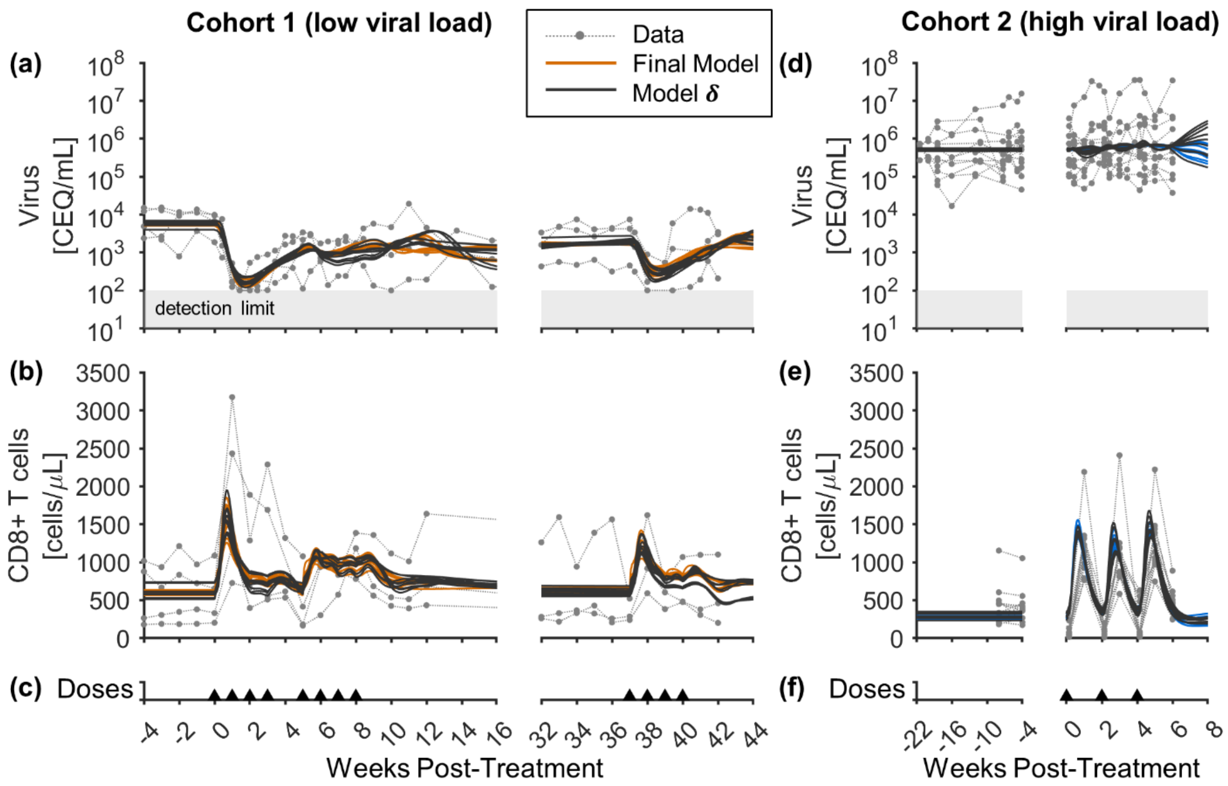

Fig E. Comparison of Model δ to Final Model. Model δ was calibrated to (a,d) virus in the plasma and (b,e) $CD8^+$ T cells in the peripheral blood from two different Simian Immunodeficiency Virus (SIV) cohorts. The top 10 results (lowest NLL, Eq. 12) from the multi-start local-search algorithm are compared for the final model (Eq. 1-11, orange/blue) and for the model δ (black). The gray shaded region indicates the lower limit of detection for the viral assay (100 CEQ/mL). Panels (c,f) show timing of 0.1 mg/kg subcutaneous doses of N-803.

Supplemental Methods

Parameter Space Discussion and Bayesian Distributions

The following is a discussion of the initial conditions in Table 1. Pre-treatment steady state viral load and $CD8^+$ T cell numbers are bounded based on the ranges of pre-treatment experimental measurements in the two NHP cohorts [6, 7]. The frequency of HIV-specific $CD8^+$ T cells in humans with HIV is variable (≈1-20%) [8-10]. We expand this range upward to account for potential differences in SIV. Since only the effect of immune regulation is relevant, the values of the regulation variables (R_1, R_2) are normalized to the pre-treatment value for Cohort 1. Thus, regulation strength parameters (φ, λ, ζ_S, ζ_N) reflect the pre-treatment effect of regulation for Cohort 1. The initial pmol/kg of N-803 at the absorption site (X) for a 0.1 mg/kg dose of N-803 is obtained from the measured molecular weight of 114 kDa [11].

The following is a discussion of the values for constants in Table 2 that govern viral infection and the $CD8^+$ T cell response. Viral growth rate (q) is based on estimates of HIV-infected cell death rate due to $CD8^+$ T cells (reviewed in [12]), since q balances the effect of $CD8^+$ T cells in the pre-treatment steady-state. The memory reversion rate (m_S) and proliferation rate (p_A) of active SIV-specific $CD8^+$ T cells reflects estimates used in $CD8^+$ T cell clonal expansion models [2, 3]. Influenza-specific memory $CD8^+$ T cell cycle in 6 hours [4] in response to antigen, which translates to a proliferation rate constant of p_A = 2.77/day. Memory reversion rate (m_N) of non-SIV-specific $CD8^+$ T cells is assumed to be faster than that of SIV-specific $CD8^+$ T cells. Active cell death rate (d_A) is sampled based on models to $CD8^+$ T cell clonal expansion [2, 3], and the resting $CD8^+$ T cell death rate (d) is based on measured turnover in healthy monkeys [13]. Proliferation stimulation factor (ρ) is limited according to a maximum drug-induced expansion rate for the SIV-naive case ($p \cdot \rho$ for this model), which is assumed to be less than the $CD8^+$ T cell clonal expansion rate in rhesus macaques (≈1/day) [14]. Other pharmacokinetic and pharmacodynamic parameters (k_a, k_e, v_d/F, C_{50}) are carried over from our previous work [15].

S1 Fig shows the distributions resulting from Bayesian MCMC for the constants of and initial conditions of Eq. 1-11.

Parameter Calculation

Prior to treatment, the system of ordinary differential equations (ODEs) (Eq. 1-11) is considered to be at an approximately steady-state for both cohorts. This is a reasonable assumption given the subjects are in the chronic phase of infection, which is reflected in the training data [6, 7]. The ODEs then become equations that relate initial conditions (Eq. S15-S23).

Eq. S15 $0 = q - \left(\frac{g\sum_{i=1}^{8} S_i}{1+\lambda R_2}\right)$

Eq. S16 $0 = P_S^* - d - A_S^* + m_S S_8/S_0$

Eq. S17 $0 = 2A_S^* S_0 - d_A S_1 - p_A S_1$

Eq. S18 $0 = 2p_A S_{i-1} - d_A S_i - p_A S_i$ where $(i \in 2,3, \dots 7)$

Eq. S19 $0 = 2p_A S_7 - d_A S_8 - m_S S_8$

Eq. S20 $0 = P_N^* - d - A_N^* + m_N N_1/N_0$

Eq. S21 $0 = 2A_N^* N_0 - d_A N_1 - m_N N_1$

Eq. S22 $0 = s_S\left(\frac{V}{V+V_{50,S}}\right) + s_N\left(\frac{V}{V+V_{50,N}}\right) - d_R R_1$

Eq. S23 $0 = R_1 - R_2$

Eq. S15-S23 and the proceeding discussion are simplified by introducing some collections (Eq. S24-S27).

Eq. S24 $P_S^* = \left(\frac{p}{1+\varphi R_2}\right)\left(\frac{S_{50}}{S_{50}+\sum_{i=0}^{8} S_i}\right)$

Eq. S25 $P_N^* = \left(\frac{p}{1+\varphi R_2}\right)\left(\frac{N_{50}}{N_{50}+N_0+N_1}\right)$

Eq. S26 $A_S^* = \left(\frac{a_S}{1+\zeta_S R_2}\right)\left(\frac{V}{V+V_{50,S}}\right)$

Eq. S27 $A_N^* = \left(\frac{a_N}{1+\zeta_N R_2}\right)\left(\frac{V}{V+V_{50,N}}\right)$

We now apply two sets of these equations (one for each cohort) to determine the model initial conditions $\{V, S_{0-8}, N_{0,1}, R_{1,2}\}$ for each cohort. Since some initial conditions, or sums thereof, are known (Table 1), some values for constants must be set so as to ensure steady-states. Special care was taken to avoid negative parameter values, but this was not foolproof. An error message was included in the model code to direct the fitting algorithm to avoiding negative points in the parameter space. In the proceeding derivation, subscripts l and h reference the low viral load cohort and high viral load cohort respectively.

Total initial SIV-specific and non-SIV-specific CD8⁺ T cells:

The total concentrations of SIV-specific CD8⁺ T cells for each cohort are obtained from the total CD8⁺ T cell concentrations ($[SN]_{\text{total}}$) and the frequencies of SIV-specific CD8⁺ T cells (f_S) (Eq. S28-S31). These are sampled for both cohorts within the values in Table 1.

Eq. S28 $$\left(\sum_{i=0}^{8} S_i\right)_l = f_{S,l}[SN]_{\text{total},l}$$

Eq. S29 $$\left(\sum_{i=0}^{8} S_i\right)_h = f_{S,h}[SN]_{\text{total},h}$$

Eq. S30 $$(N_0 + N_1)_l = \left(1 - f_{S,l}\right)[SN]_{\text{total},l}$$

Eq. S31 $$(N_0 + N_1)_h = \left(1 - f_{S,h}\right)[SN]_{\text{total},h}$$

50% resting proliferation concentrations: S_{50}, N_{50}

To reduce the parameter space, we sampled the sum of the concentrations for 50% resting proliferation, (S_{50}+N_{50}), and calculated expected 50% proliferation concentrations, S_{50}, N_{50}, using the fraction of SIV-specific cells for cohort 1, $f_{S,l}$ (Eq. S32,S33).

Eq. S32 $$S_{50} = f_{S,l}(S_{50}+N_{50})$$

Eq. S33 $$N_{50} = \left(1 - f_{S,l}\right)(S_{50}+N_{50})$$

Regulation initial values and generation parameters: $[\boldsymbol{R}, \boldsymbol{s_S}, \boldsymbol{s_N}]$

Regulation is normalized to the low-VL group ($R_l = 1$). Regulation for the high-VL group, R_h, is calculated (Eq. S34), where the low and high initial viral loads are V_l and V_h and where the ratio s_N/s_S is sampled.

Eq. S34 $$R_h = \frac{\left(\frac{V_h}{V_h+V_{50,S}}\right)+\left(\frac{s_N}{s_S}\right)\left(\frac{V_h}{V_h+V_{50,N}}\right)}{\left(\frac{V_l}{V_l+V_{50,S}}\right)+\left(\frac{s_N}{s_S}\right)\left(\frac{V_l}{V_l+V_{50,N}}\right)} \quad \text{where } \left(\frac{s_N}{s_S}\right) \in [0.1,100] \text{ (Table 2)}$$

Regulation generation parameters, s_S, s_N, can be calculated from either cohort (Eq. S35,S36).

Eq. S35 $$s_S = d_R / \left[\left(\frac{V_l}{V_l+V_{50,S}}\right) + \left(\frac{s_N}{s_S}\right)\left(\frac{V_l}{V_l+V_{50,N}}\right)\right]$$

Eq. S36 $$s_N = \left(\frac{s_N}{s_S}\right) s_S$$

Activation rate constants and regulation factors: $[\boldsymbol{a_S}, \boldsymbol{a_N}, \boldsymbol{\zeta_S}, \boldsymbol{\zeta_N}]$

Relationships between $A_S^* S_0$ and S_8 (Eq. S37) and between $A_N^* N_0$ and N_1 (Eq. S38) can be derived from Eq. S17-S19,S21. These apply to both cohorts.

Eq. S37 $U_S = \frac{S_8}{S_0 A_S^*} = \frac{2}{d_A + m_S}\left(\frac{2p_A}{d_A + p_A}\right)^7$

Eq. S38 $U_N = \frac{N_1}{N_0 A_N^*} = \frac{2}{d_A + m_N}$

The following equations are written with respect to SIV-specific CD8+ T cells, S, but analogous equations apply to non-SIV-specific CD8+ T cells, N. Equation S16 can now be rewritten (Eq. S39) to obtain initial activation rates A_S^* from initial proliferation rates P_S^*.

Eq. S39 $0 = P_S^* - d + (m_S U_S - 1)A_S^*$

The following (Eq. S40,S1) obtain a_S and ζ_S from the values of A_S^* for each cohort.

Eq. S40 $\zeta_S = \frac{\Omega_S - 1}{R_h - \Omega_S}$ where $\Omega_S = \frac{A_{S,l}^*}{A_{S,h}^*}\left(\frac{V_h}{V_l}\right)\left(\frac{V_l + V_{50,S}}{V_h + V_{50,S}}\right)$

Eq. S41 $a_S = A_{S,l}^*\left(\frac{V_l + V_{50,S}}{V_l}\right)(1 + \zeta_S)$

<u>Initial conditions for CD8+ T cell subgroups</u>: $[\boldsymbol{S_{0,\ldots 8}}, \boldsymbol{N_{0,1}}]$

With values for A_S^* and A_N^* now known, CD8+ T cell subgroups follow from Eq. S17-S19,S21 and from known values for total S and N.

<u>Killing rate constant and regulation factor</u>: $[\boldsymbol{g}, \boldsymbol{\lambda}]$

If the killing regulation factor (λ) and the killing rate constant g are obtained by ensuring that the viral equation, Eq. S15, is satisfied for both cohorts.

Eq. S42 $\lambda = \frac{(\sum_{i=1}^{8} S_i)_h - (\sum_{i=1}^{8} S_i)_l}{(\sum_{i=1}^{8} S_i)_l R_h - (\sum_{i=1}^{8} S_i)_h}$

Eq. S43 $g = q \frac{1 + \lambda}{(\sum_{i=1}^{8} S_i)_l}$

Fitting to single cohorts (for S3 Fig)

The multi-start local-search fitting process is repeated for each cohort individually to see if substantively better fits could be obtained. With only one pre-treatment steady-state to consider, fewer constants have to be calculated. Constants that were sampled instead of calculated are summarized in Table D. Results of calibration with and without shared cohort parameters are compared in S3 Fig. The most noteworthy difference is in the CD8⁺ T cell expansion in cohort 1 (S3B Fig), though both methods agree reasonably with the data.

Table D. Newly sampled constants for Single Cohort Fitting

Initial Condition or Parameter	Symbol	Value
Regulation factor for activation (ST8,NT8)	ζ_S, ζ_N	(0.01, 100)
Regulation factor for killing	λ	(0.01, 100)

Comparison to other non-human primate cohorts (for S4 Fig)

The model was also compared to intravenously administered N-803 in SIV-naïve and SIV-infected non-human primates [16]. To model intravenous administration, doses were applied directly to the bioavailable compartment. Thus, X_0/v_d became the initial condition for the compartment, which decayed at the same elimination rate (k_e, Eq. S44).

Eq. S44 $$C' = -k_e C$$

To model the SIV-naive case (S4A Fig), SIV virions (V, Eq. 1) and SIV-specific CD8⁺ T cells (S_0-S_8, Eq. 2-5) were absent. Also, active non-SIV-specific CD8⁺ T cells (N_1) and immune regulation (R_1, R_2) were initially zero but could still be induced by N-803. Thus, resting non-SIV-specific CD8⁺ T cells (N_0) composed the entire CD8⁺ T cell pool prior to treatment. The pre-treatment steady-state of these cells was calculated from parameters (Eq. S45). Thus, initial conditions in the model do not directly match the initial values in the validation data, as this was necessary to have a pre-treatment steady-state using the same parameter sets calibrated to Cohort 1 [6] and Cohort 2 [7].

Eq. S45 $$N_0 = N_{50}(p/d - 1)$$

S4A Fig shows the CD8⁺ T cells in the blood of SIV-naive NHPs (n=4) after a 6 mg/kg intravenous dose of N-803 [16]. There is a brief drop in CD8⁺ T cells at day 1. This could be due to extravasation [17, 18], which

that our model does not incorporate. Otherwise, both model and data show a modest expansion of $CD8^+$ T cells. This can be compared to the larger expansion after an identical dose given to SIV-infected NHPs (also n=4, S4D Fig). S4D Fig also shows the results of two additional 6 mg/kg intravenous doses given at 1 and 2 weeks after the first dose, as well as a large 100 mg/kg dose at week 7. Both model and data show smaller expansions after the two subsequent 6 mg/kg doses than after the first dose (week 0). Model and data seem to diverge for the 100 mg/kg dose, where the model predicted a weaker expansion than is observed in the data. This could be due to the reduced viral load in the model (S4C Fig).

S4C Fig shows the response of viral load for one subject after the 100 mg/kg intravenous dose of N-803 [16], being the only subject with viral load data that was above the detection limit. Both model and data show an approximately 10-fold reduction in viral load following this dose, and both return to the pre-dose state after a brief period. Thus, our model reproduces several key qualitative aspects of an N-803 data set with an independent NHP cohort, different disease state (SIV-naïve vs. SIV-infected), and different route of N-803 delivery (intravenous vs. subcutaneous).

Reference

1. Bedrick EJ, Tsai C-L. Model Selection for Multivariate Regression in Small Samples. Biometrics. 1994;50(1):226-31. doi: 10.2307/2533213.
2. Wick WD, Yang OO. Biologically-directed modeling reflects cytolytic clearance of SIV-infected cells in vivo in macaques. PloS one. 2012;7(9):e44778. Epub 2012/10/03. doi: 10.1371/journal.pone.0044778. PubMed PMID: 23028619.
3. Jones LE, Perelson AS. Transient viremia, plasma viral load, and reservoir replenishment in HIV-infected patients on antiretroviral therapy. Journal of acquired immune deficiency syndromes (1999). 2007;45(5):483-93. Epub 2007/05/15. doi: 10.1097/QAI.0b013e3180654836. PubMed PMID: 17496565.
4. Lalvani A, Brookes R, Hambleton S, Britton WJ, Hill AV, McMichael AJ. Rapid effector function in CD8+ memory T cells. The Journal of experimental medicine. 1997;186(6):859-65. Epub 1997/09/18. doi: 10.1084/jem.186.6.859. PubMed PMID: 9294140.
5. Kim J, Chang DY, Lee HW, Lee H, Kim JH, Sung PS, et al. Innate-like Cytotoxic Function of Bystander-Activated CD8(+) T Cells Is Associated with Liver Injury in Acute Hepatitis A. Immunity. 2018;48(1):161-73.e5. Epub 2018/01/07. doi: 10.1016/j.immuni.2017.11.025. PubMed PMID: 29305140.
6. Ellis-Connell AL, Balgeman AJ, Zarbock KR, Barry G, Weiler A, Egan JO, et al. ALT-803 Transiently Reduces Simian Immunodeficiency Virus Replication in the Absence of Antiretroviral Treatment. Journal of virology. 2018;92(3). Epub 2017/11/10. doi: 10.1128/jvi.01748-17. PubMed PMID: 29118125.
7. Ellis-Connell AL, Balgeman AJ, Harwood OE, Moriarty RV, Safrit JT, Weiler AM, et al. Control of Simian Immunodeficiency Virus Infection in Prophylactically Vaccinated, Antiretroviral Treatment-Naive Macaques Is Required for the Most Efficacious CD8 T Cell Response during Treatment with the Interleukin-15 Superagonist N-803. Journal of virology. 2022;96(20):e0118522. Epub 2022/10/04. doi: 10.1128/jvi.01185-22. PubMed PMID: 36190241.

8. Betts MR, Ambrozak DR, Douek DC, Bonhoeffer S, Brenchley JM, Casazza JP, et al. Analysis of total human immunodeficiency virus (HIV)-specific CD4(+) and CD8(+) T-cell responses: relationship to viral load in untreated HIV infection. Journal of virology. 2001;75(24):11983-91. Epub 2001/11/17. doi: 10.1128/jvi.75.24.11983-11991.2001. PubMed PMID: 11711588.

9. Migueles SA, Connors M. Frequency and function of HIV-specific CD8(+) T cells. Immunology letters. 2001;79(1-2):141-50. Epub 2001/10/12. doi: 10.1016/s0165-2478(01)00276-0. PubMed PMID: 11595301.

10. Gea-Banacloche JC, Migueles SA, Martino L, Shupert WL, McNeil AC, Sabbaghian MS, et al. Maintenance of large numbers of virus-specific CD8+ T cells in HIV-infected progressors and long-term nonprogressors. Journal of immunology (Baltimore, Md : 1950). 2000;165(2):1082-92. Epub 2000/07/06. doi: 10.4049/jimmunol.165.2.1082. PubMed PMID: 10878387.

11. Han KP, Zhu X, Liu B, Jeng E, Kong L, Yovandich JL, et al. IL-15:IL-15 receptor alpha superagonist complex: high-level co-expression in recombinant mammalian cells, purification and characterization. Cytokine. 2011;56(3):804-10. Epub 2011/10/25. doi: 10.1016/j.cyto.2011.09.028. PubMed PMID: 22019703.

12. Gadhamsetty S, Beltman JB, de Boer RJ. What do mathematical models tell us about killing rates during HIV-1 infection? Immunology letters. 2015;168(1):1-6. Epub 2015/08/19. doi: 10.1016/j.imlet.2015.07.009. PubMed PMID: 26279491.

13. De Boer RJ, Mohri H, Ho DD, Perelson AS. Turnover rates of B cells, T cells, and NK cells in simian immunodeficiency virus-infected and uninfected rhesus macaques. Journal of immunology (Baltimore, Md : 1950). 2003;170(5):2479-87. Epub 2003/02/21. doi: 10.4049/jimmunol.170.5.2479. PubMed PMID: 12594273.

14. Davenport MP, Ribeiro RM, Perelson AS. Kinetics of virus-specific CD8+ T cells and the control of human immunodeficiency virus infection. Journal of virology. 2004;78(18):10096-103. Epub 2004/08/28. doi: 10.1128/jvi.78.18.10096-10103.2004. PubMed PMID: 15331742.

15. Cody JW, Ellis-Connell AL, O'Connor SL, Pienaar E. Mathematical modeling of N-803 treatment in SIV-infected non-human primates. PLoS computational biology. 2021;17(7):e1009204. Epub 2021/07/29. doi: 10.1371/journal.pcbi.1009204. PubMed PMID: 34319980.

16. Webb GM, Li S, Mwakalundwa G, Folkvord JM, Greene JM, Reed JS, et al. The human IL-15 superagonist ALT-803 directs SIV-specific CD8(+) T cells into B-cell follicles. Blood advances. 2018;2(2):76-84. Epub 2018/01/25. doi: 10.1182/bloodadvances.2017012971. PubMed PMID: 29365313.

17. Weninger W, Crowley MA, Manjunath N, von Andrian UH. Migratory properties of naive, effector, and memory CD8(+) T cells. The Journal of experimental medicine. 2001;194(7):953-66. Epub 2001/10/03. doi: 10.1084/jem.194.7.953. PubMed PMID: 11581317.

18. Sowell RT, Goldufsky JW, Rogozinska M, Quiles Z, Cao Y, Castillo EF, et al. IL-15 Complexes Induce Migration of Resting Memory CD8 T Cells into Mucosal Tissues. Journal of immunology (Baltimore, Md : 1950). 2017;199(7):2536-46. Epub 2017/08/18. doi: 10.4049/jimmunol.1501638. PubMed PMID: 28814601.
