# Supplementary figures and images for: Mathematical modeling indicates that regulatory inhibition of CD8+ T cell cytotoxicity can limit efficacy of IL-15 immunotherapy in cases of high pre-treatment SIV viral load
Source: PLoS Comput Biol. 2023 Aug 24;19(8):e1011425. doi: 10.1371/journal.pcbi.1011425 (PMC10482305; doi:10.1371/journal.pcbi.1011425)

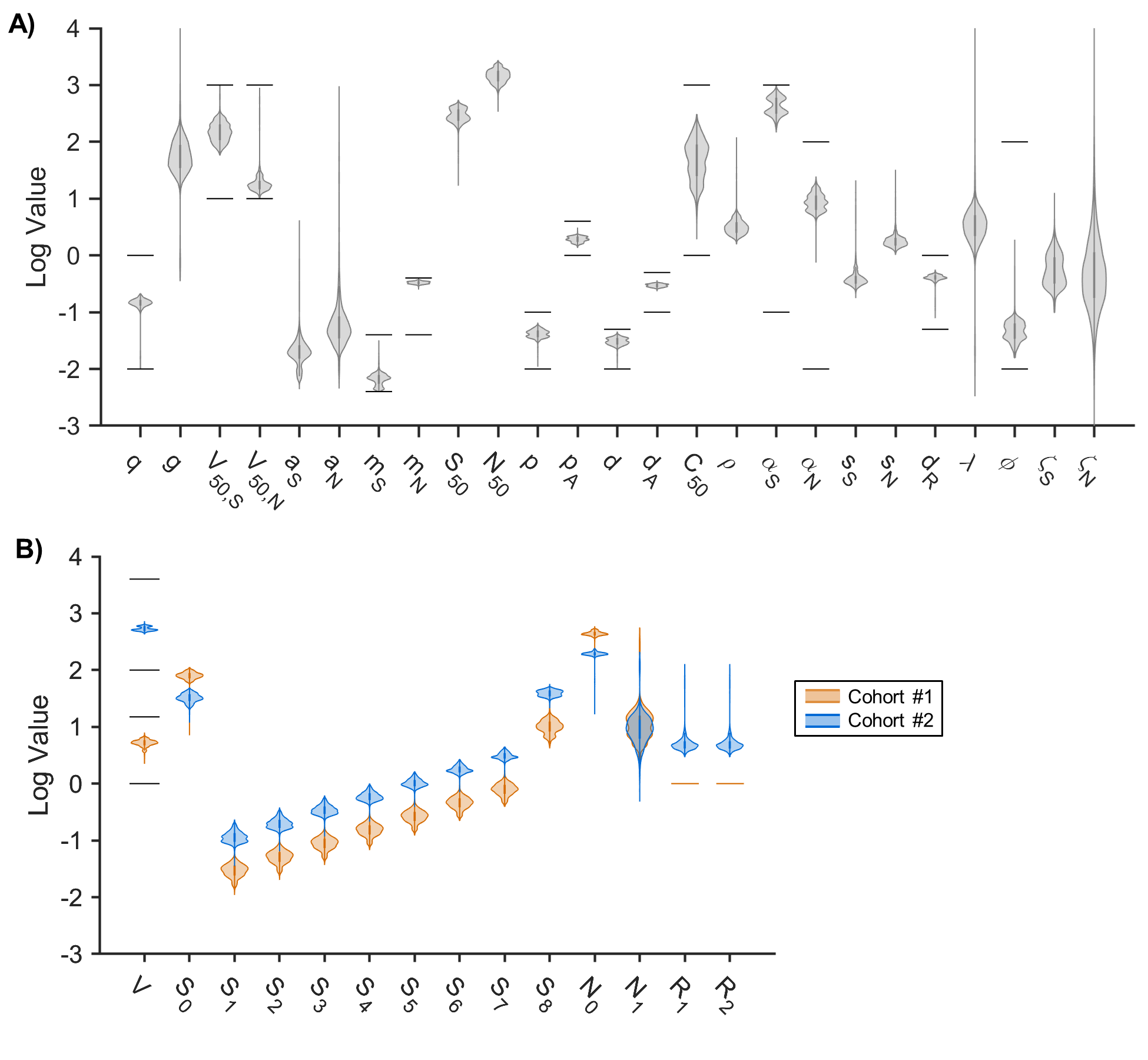

Supplement: S1 Fig — Shown is the Bayesian MCMC sample of values for constants (A) and initial conditions (B) in Eqs 1–11. Solid lines indicate allowed ranges, which are also given in Tables 1 and 2. Constants and initial conditions without ranges are calculated by assuming pre-treatment steady-state or by sampling ratios relative to other parameters. Units are as given in Table 2, except that g is converted to nL/#/d. (TIF) [file pcbi.1011425.s002.tif]

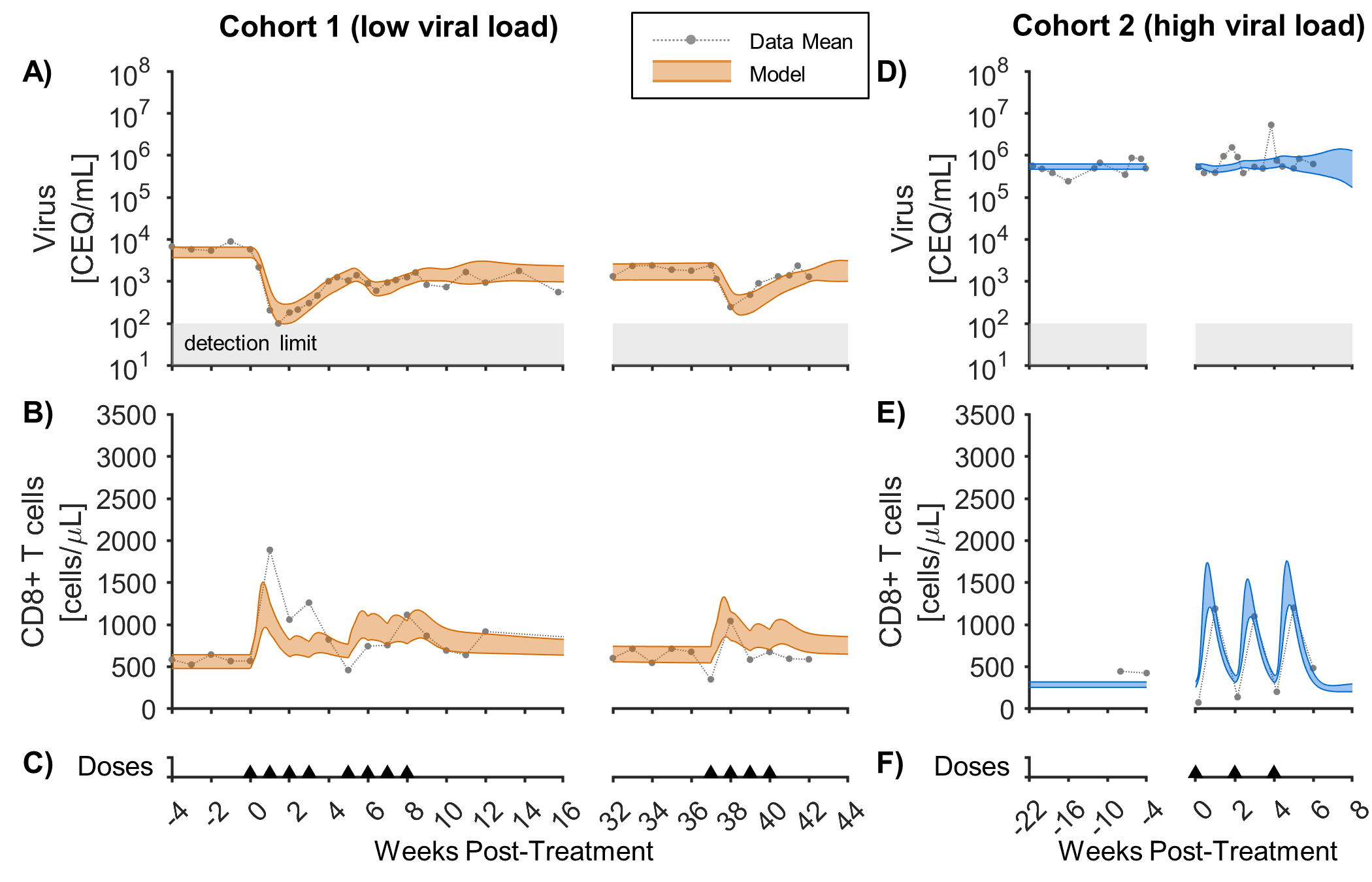

Supplement: S2 Fig — This is a repeat of Fig 3, but where the model is instead compared to the mean of the data points, across each cohort, at each time point. The model was calibrated to (A,D) virus in the plasma and (B,E) CD8+ T cells in the peripheral blood from two different Simian Immunodeficiency Virus (SIV) cohorts. The orange and blue shaded regions correspond to the Bayesian 95% credible interval of the mathematical model. The gray shaded region indicates the lower limit of detection for the viral assay (100 CEQ/mL). Panels (C,F) show timing of 0.1 mg/kg subcutaneous doses of N-803. (TIF) [file pcbi.1011425.s003.tif]

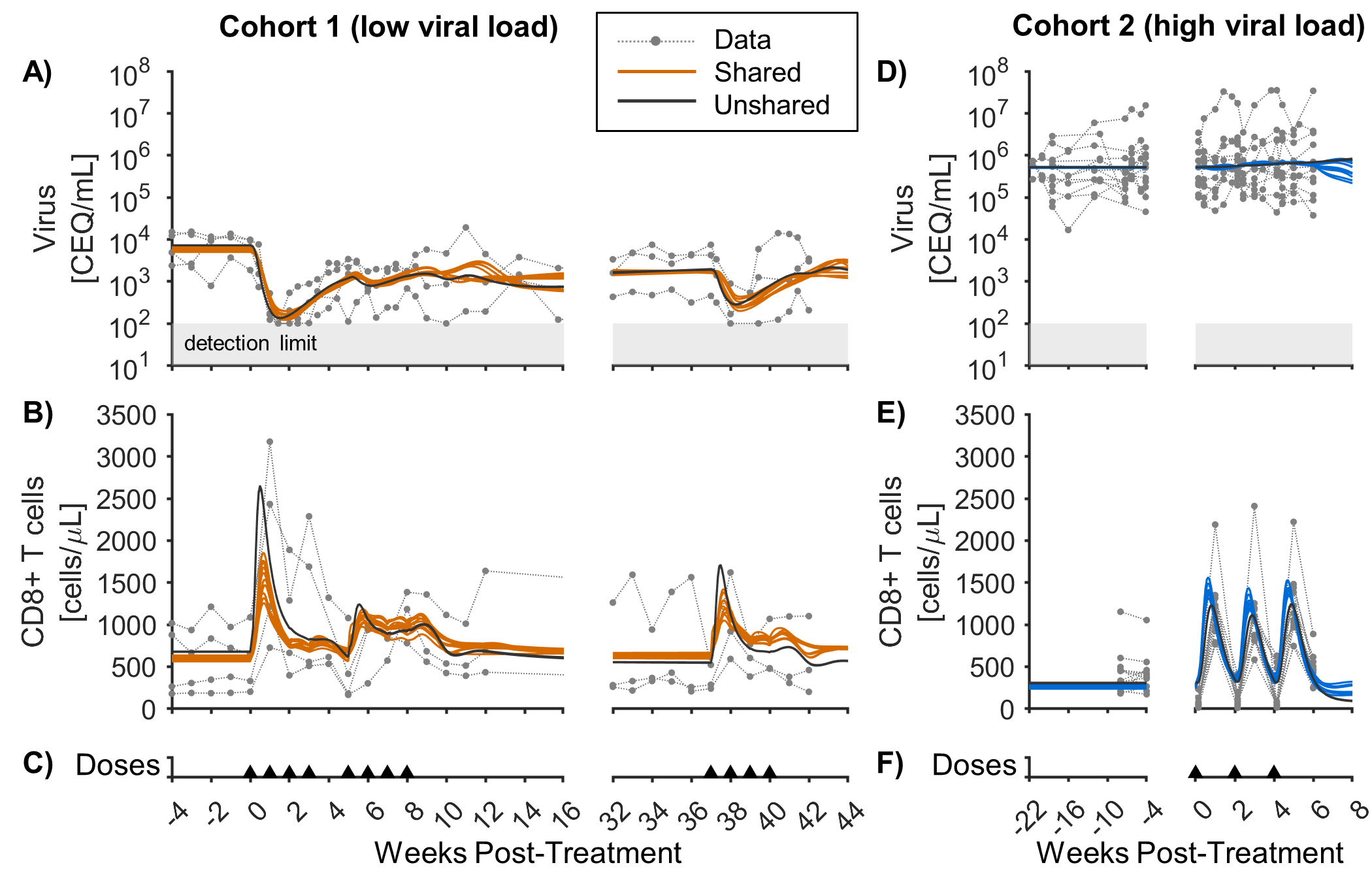

Supplement: S3 Fig — The model was calibrated to (A,D) log fold change in virus in the plasma and (B,E) fold change in CD8+ T cells in the peripheral blood from two different Simian Immunodeficiency Virus (SIV) cohorts. Shown are top 10 results (lowest NLL) from the multi-start local-search algorithm for two different scenarios. The orange/blue lines correspond to the scenario in Fig 3, where the same set of model constants is used for both cohorts (“Shared”). The black lines correspond the case where different sets of constants are fitted to each cohort (“Unshared”). The gray shaded region indicates the lower limit of detection for the viral assay (100 CEQ/mL). Panels (C,F) show timing of 0.1 mg/kg subcutaneous doses of N-803. See S1 Text for additional methodological details. (TIF) [file pcbi.1011425.s004.tif]

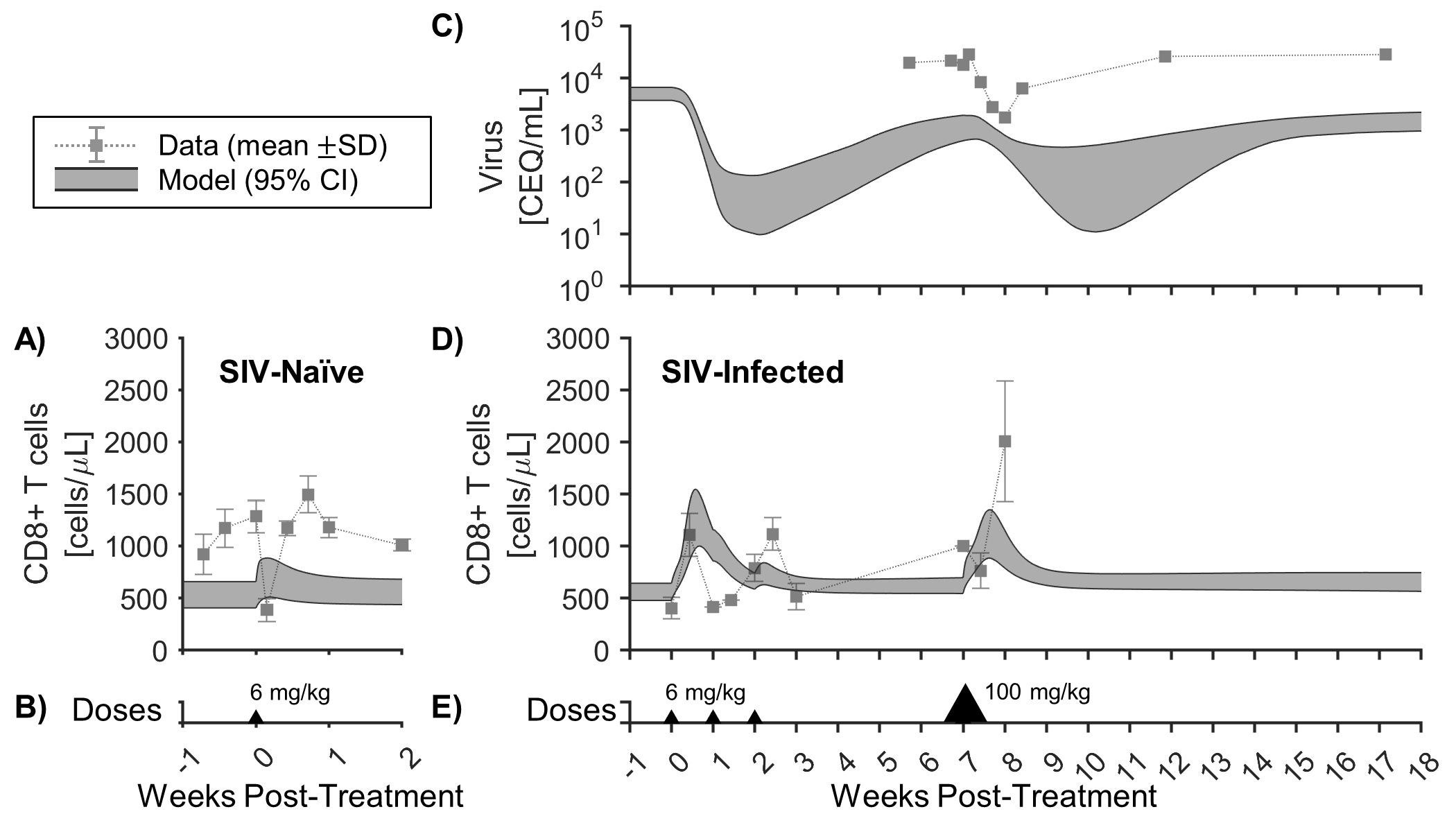

Supplement: S4 Fig — Model predictions are compared to a different SIV NHP cohort [10]. In panel A, SIV-naïve NHPs (n = 4) are given a 6 mg/kg intravenous dose of N-803 at week 0. In panel D, SIV-infected NHPs (n = 4) are given three 6 mg/kg doses spaced one week apart, followed by a 100 mg/kg dose 5 weeks later. Peripheral blood CD8+ T cell counts are shown in both panels (mean and standard deviation) and compared to mathematical model predictions (Bayesian 95% credible interval). Panel C shows the plasma viral load after the 100 mg/kg dose (one data subject compared to model prediction). NHP data was obtained from published figures using Engage Digitizer software. Panels (B,E) show timing and size of intravenous doses of N-803. See S1 Text for additional methodological details and discussion of results. (TIF) [file pcbi.1011425.s005.tif]

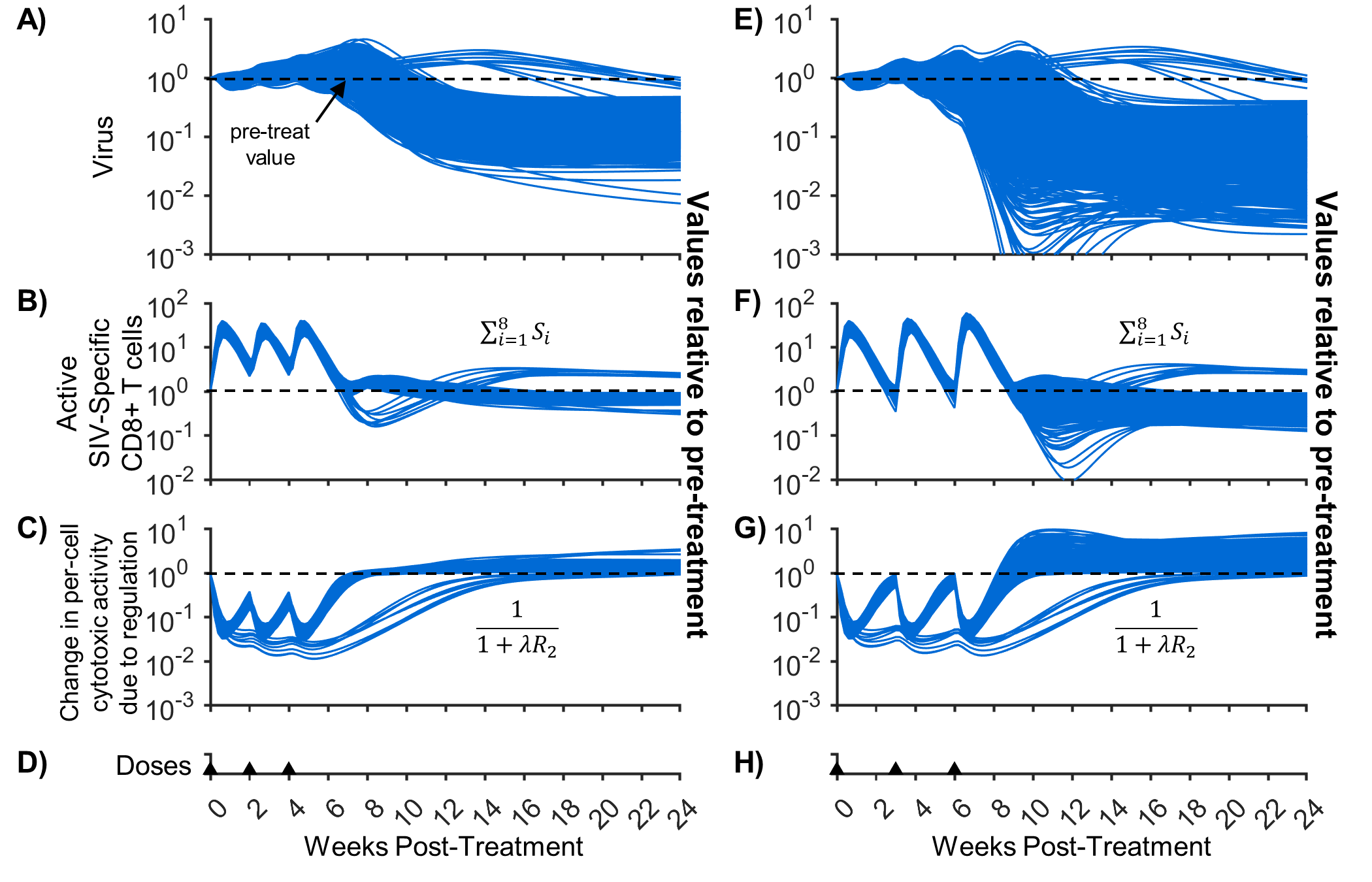

Supplement: S5 Fig — Shown are the model predicted responses of the high viral load cohort using a subset of 1000 parameter sets from the Bayesian MCMC parameter sample, representing a simulated cohort. Panels A-D and E-H correspond to 3 doses given either 2 or 3 weeks apart, respectively (the 2-week regimen being the original regimen). Panels A,E show the viral load, panels B,F show the total active SIV-specific CD8+ T cells, and panels C,G show the change in cytotoxicity due to regulatory inhibition. Equivalent terms from Eqs 1–11 are shown within each axis. Values for each fitted parameter set were normalized to the Cohort 2 pre-treatment baseline for that parameter set (marked by dashed line. Panels D,H show the timing of 0.1 mg/kg subcutaneous doses of N-803. (TIF) [file pcbi.1011425.s006.tif]

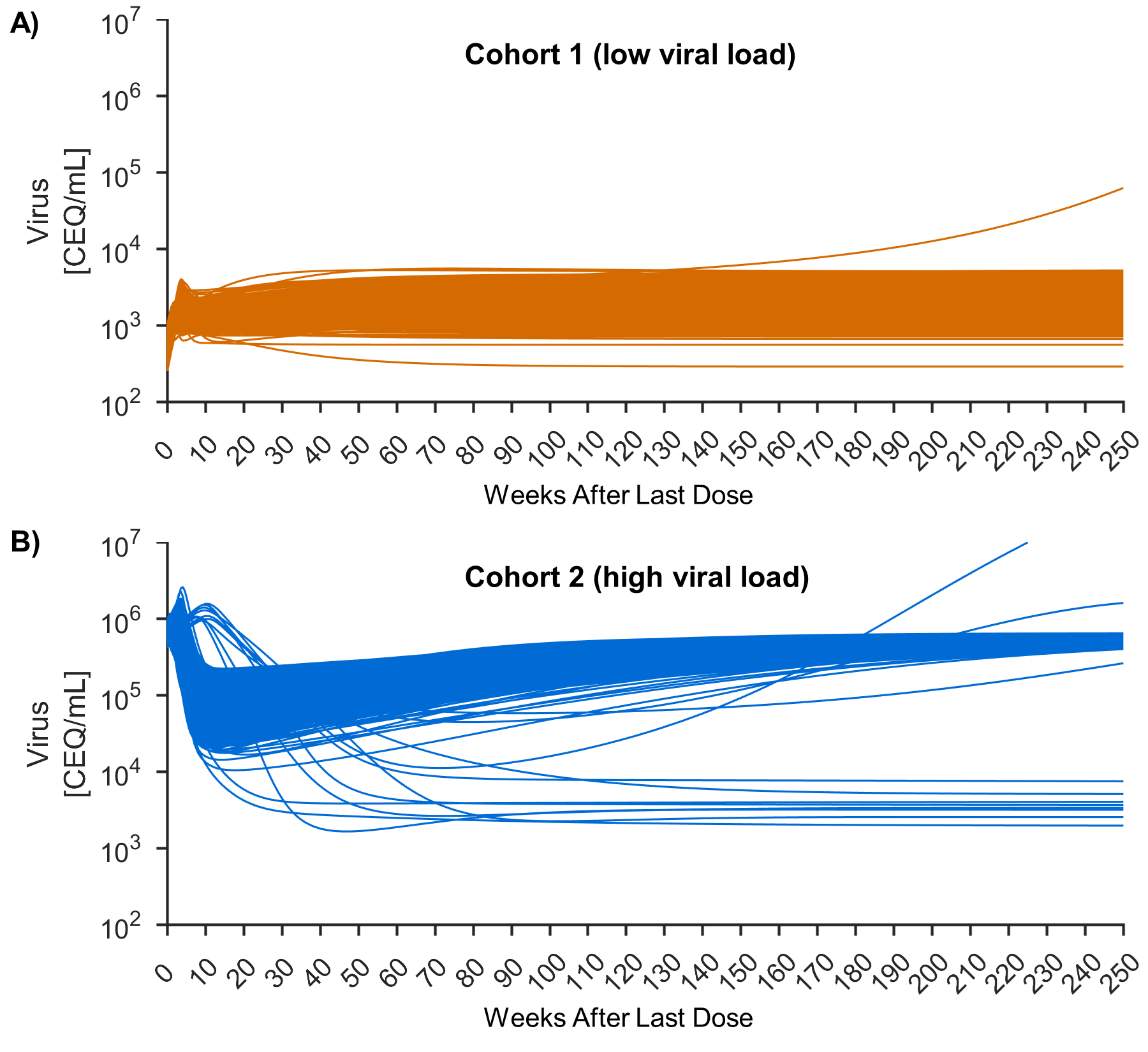

Supplement: S6 Fig — Shown are long-term model projections after the last N-803 is given to low viral load cohort (panel A) and high viral load cohort (panel B) using a subset of 1000 parameter sets from the Bayesian MCMC parameter sample. (TIF) [file pcbi.1011425.s007.tif]
