# Supplementary material for: Mathematical modeling indicates that regulatory inhibition of CD8+ T cell cytotoxicity can limit efficacy of IL-15 immunotherapy in cases of high pre-treatment SIV viral load
Source: PLoS Comput Biol. 2023 Aug 24;19(8):e1011425. doi: 10.1371/journal.pcbi.1011425 (PMC10482305; doi:10.1371/journal.pcbi.1011425)
Supplement: Attachment — Submitted filename: Response to Reviewers.pdf [file pcbi.1011425.s009.pdf]

First, we would like to apologize for any confusion caused by the original response document. We had mistakenly misattributed reviewer comments in that document. Reviewer comments below are now listed in the order they were received. ***Our responses are in bold below each comment.*** *Descriptions of unsolicited changes are moved to the end of the document.*

Reviewer 1

In the article titled "mathematical modeling indicates that regulatory inhibition of CD8+ T cell cytotoxicity can limit efficacy of IL-15 immunotherapy in cases of high pre-treatment SIV viral load", the authors built on their previous work to study the driving factor behind the low efficacy of IL-15 treatment for SIV infected non-human primate. As the title suggests, the main finding is that IL-15 treatment is limited due to regulatory inhibition of CD8+ T cells in non-human primates with high pre-treatment viral load. The study is well-motivated and the result is intuitive, well-reasoned and demonstrated. The technical aspects, such as model parametrization, are strong. The entire procedure seems clearly presented and should be reproducible. I only have a few minor comments regarding the manuscript.

1. I find the model somewhat unnecessarily complicated. For example, does including S1 to S8 compartments for the different activated stages of CD8+ T cells necessarily improve model fit, because it does not seem to affect any other aspect of the manuscript? Have the authors tried to simplify the models?

Following the reviewers' and editor's suggestions, we evaluated many alternative formulations using Akaike Information Criterion (AIC), which resulted in a slightly simplified model. Now non-SIV-specific $CD8^+$ T cells (Eq. 7, line 173) only divide once after activation and their contribution to SIV suppression is not explicitly included (Eq. 1, line 167). These changes eliminated three parameters and reduced the AIC compared to the previous model. The SIV-specific cell equations were not changed, but we included calibrations of four alternative models that justify our chosen model. These are discussed in the first section of S1 Appendix. All of these qualitatively replicate our results, but they are inferior to the chosen model, based on AIC comparison (Figure S1) . We believe that these model simplifications improve the robustness of our model and of our findings, which still hold with this simplified model.

2. While the methods are clear, it is still rather convoluted. I think having a flow-chart figure showing the steps taken for model parametrization would be helpful. Additionally, fitting is done on individual patients simultaneously (Eq. S38-39), so it seems including a plot with the fit and the average of the data would be necessary, especially because the 95% credible interval misses the high and low points due to individual heterogeneity (Fig. 1).

We added Figure 2, which is a flowchart showing our analytical approach. We also moved our description of analytical methods from the supplement to the main text (Parameter Estimation section), as well as improved clarification and summarization of the process. We also took steps to make the parameter calculation less complex, which should be reflected in Tables 2 and 3, as well as the Parameter Calculation section in S1 Appendix. We also included comparison to the mean of data in S1 Appendix (Figure S7).

[continuation of 2.] Another point is that the 95% credible interval for cohort 2 seems to indicate a decreasing trend near the end – week 8 post treatment - (Fig. 1D), which corresponds to an increasing CD8+ T cells (Fig. 1E). Since the model dynamics is not known analytically, extending the simulation to check whether this declining trend leads to viral extinction is necessary. I bring up this point because of the SIV dynamic (Eq. 1) can be written as V' = (…)V, so if the terms in (…) is negative, SIV may become extinct.

The new treatment exploration figure (Fig 6) includes projections of viral load after the treatment regimen. We also include long-term plots of the model behavior for both cohorts after treatment in S1 Appendix (Fig S11). While viral load never becomes extinct, there are parameter sets where it decays to a set point close to cohort 1's pre-treatment state. The mechanisms driving this post-treatment decline are described on lines 468-479.

3. After MCMC to obtain posterior distributions of the initials, are the mean used for subsequent fitting and analysis? I think this information is missing from the manuscript. However, if this is the case, there is another potential issue. The study looks at the "average" of the population to avoid individual heterogeneity; however, for such a complicated models, chaotic behaviors are not ruled out. Thus, infinitesimally small difference in initials can lead to completely different dynamical behavior qualitatively. And because the parameter distribution of the initials are so wide (some vary over 4-5 logs in value), this point should be addressed for the results to be credible.

The reviewer is partly correct. We were holding viral load and total $CD8^+$ T cell initial conditions fixed for both cohorts based on the averages of the experimental data, while the initial conditions for regulation and $CD8^+$ T cell subgroups were sampled using the Bayesian MCMC. In our revised manuscript, we now allow those previously fixed quantities to vary. We also included initial condition distributions in Fig S6 (in S1 Appendix). This update did not affect our conclusions.

4. Some minor preferences. I find the term "validated" a bit too strong. Something like "tested" or "supported" maybe more suitable.

We now use 'compare to', and similar language, to describe model comparisons with independent data sets, rather than 'validate against'.

[continuation of 4.] Also, while the authors frame the differences in the initials (pre-treatment viral load, etc.) "can influence immunotherapeutic efficacy" (abstract), the results show the underlying differences (differences in parameters) are the reason behind this. On this note, please clarify the statement on lines 433-434.

Our apologies, but we may not have communicated this clearly. Each instance of the model is run once for each cohort using the same set of constants but different initials. The discussion on lines 431-434 (now lines 526-529) is referring to this, that one can get two different treatment results using the same constants but starting from different points. We updated our methods descriptions to make this more clear, including adding the Figure 2 flowchart. We also improved the precision of our language to use the terms 'constants' and 'initials', with 'parameters' being reserved for a combined set of constants and initials.

Reviewer 2

The paper uses a mathematical model and two cohort data to determine virus-host differences in subjects that respond to an IL-15 antagonist therapy and the ones who do not. The idea is interesting, but the presentation is very dense and the model needs further justification and explanation. I will detail my suggestions below.

1. The model is very large, with 20 equations for immune activation following drug initiation. It is adapted from previous studies and not completely explained here. That makes it hard to follow. In particular:

a. Why do you need an antigen dependent and antigen independent expansion of T cells?

b. Why do you use 7 classes in the S expansion? Is that number relevant, or fewer classes will suffice?

Following the reviewers' and editor's suggestions, we evaluated many alternative formulations using Akaike Information Criterion (AIC), which resulted in a slightly simplified model. Now non-SIV-specific CD8^{+} T cells (Eq. 7, line 173) only divide once after activation and their contribution to SIV suppression is not explicitly included (Eq. 1, line 167). These changes eliminated three parameters and reduced the AIC compared to the previous model. The SIV-specific cell equations were not changed, but we included calibrations of four alternative models that justify our chosen model. These are discussed in the first section of S1 Appendix. All of these qualitatively replicate our results, but they are inferior to the chosen model, based on AIC comparison (Figure S1) . We believe that these model simplifications improve the robustness of our model and of our findings, which still hold with this simplified model.

c. Why do you use an antigen class and not the virus class V for immune activation? You can apply the drug effect directly into the V equation.

There may have been some confusion due to how our equations were represented. Equations for activation and proliferation rate modifiers A_S, A_N, and P were algebraic, not differential. They were included to clean up the differential equations, as those terms appear in multiple places. We now inserted those terms into the differential equations directly (Eq. 2,3,6,7,10, lines 168-176).

d. Why did you not consider a model of viral infection (with target cells and infected cells)?

This is an excellent question, and we have considered a model with target and infected cells. We agree that it would make a more robust and stable model, but we concluded that it was not justified by the data that we had available. We have now elaborated on this assumption on lines 181-188.

e. You used quasi-equilibrium assumptions during data fitting. Can you reduce the model taking those into consideration?

We tested condensing the delays regarding CD8^{+} T cell activation, expansion, and regulation in our analysis described under point 'b' above.

2. I have several issues on data fitting. In particular:

a. Can you explain how you fit the population to data on fold changes rather than concentration values.

We now decided to instead fit to the concentration values directly. This made our results figures easier to interpret, but did not change any conclusions.

b. It is not clear which parameters are being fitted (especially in the main manuscript). Please explain that.

We elaborated on this in the captions for Tables 1 and 2.

c. What are the differences between the three data symbols? It was hard to follow which ones were below limit of detection. List how many data points you use for data fitting, that way one can compare number of parameters being estimated to number of data points.

The different data symbols referred to different individual NHPs, though we did mistakenly use only three symbols. We have opted now to just use the same symbol for each individual, to reduce clutter. We also add a shade region marking the detection limit in Figure 3. We included number of data points for each data set in Table 3.

d. Did you compute an AIC for the model after data fitting?

We used AIC to compare model alternatives. Results of comparisons are now included in S1 Appendix.

3. Can you use the results from cohort 1 to explain the low initial data subjects in cohort 2?

This is a good question and would be a useful application/extrapolation of our model. However, we do not believe that it would be prudent to make conclusions for this dataset. Since their experimentally

measured viral loads are so close to the limit of detection, we really do not know what response these monkeys had to the treatment, so there would be no robust way to evaluate our predictions.

4. Can you use the model to design in-silico experiments that can tell you under what conditions the drugs will work for the high initial titer? Such as using different dosing or timings?

This is an excellent suggestion. We now have added a new results section that tests different dose schedules for cohort 2 (beginning line 456).

5. Minor issues:

a. The use of p and P in the same equation, same with a and A.

b. the sum of S+N should have summation indexes.

c. Add A and H into the variables table.

All of these presentation issues were addressed. Algebraic expressions for A_S, A_N, and P were inserted into their corresponding differential equations. Note that H, which was a density-dependent proliferation parameter, has been separated into two parameters S_{50} and N_{50}. (See the note at the bottom of this document.)

Data and code availability

Have the authors made all data and (if applicable) computational code underlying the findings in their manuscript fully available?

Reviewer 1: "The authors refer to other publications for data set. No explicit data set is given."

Reviewer 2: "I did not see any code or data being shared within the manuscript of SI."

We did include a link to a GitHub release in S1 Appendix. We updated that release, and it is now referenced in the main text at the beginning of the Methods section (lines 127-128). This includes all data and code necessary to replicate results.

Unsolicited changes

Evaluation of model stability for a follow-up paper revealed that our density-dependent proliferation terms were not behaving as intended (H terms in the original model, now S_{50},N_{50} terms in Eq. 2,6 lines 168,172). These terms now only sum either SIV-specific or non-SIV-specific $CD8^+$ T cells in the denominator, rather than total $CD8^+$ T cells. This did not affect our results for this paper.
